# Specific Ion Effects on Aggregation and Charging Properties of Boron Nitride Nanospheres

**DOI:** 10.1021/acs.langmuir.0c03533

**Published:** 2021-02-08

**Authors:** Tímea Hegedűs, Dóra Takács, Lívia Vásárhelyi, István Szilágyi, Zoltán Kónya

**Affiliations:** † Department of Applied and Environmental Chemistry, University of Szeged, Szeged H-6720, Hungary; ‡ MTA-SZTE Lendület Biocolloids Research Group, Interdisciplinary Excellence Center, Department of Physical Chemistry and Materials Science, University of Szeged, H-6720 Szeged, Hungary; § MTA-SZTE Reaction Kinetics and Surface Chemistry Research Group, Szeged H-6720, Hungary

## Abstract

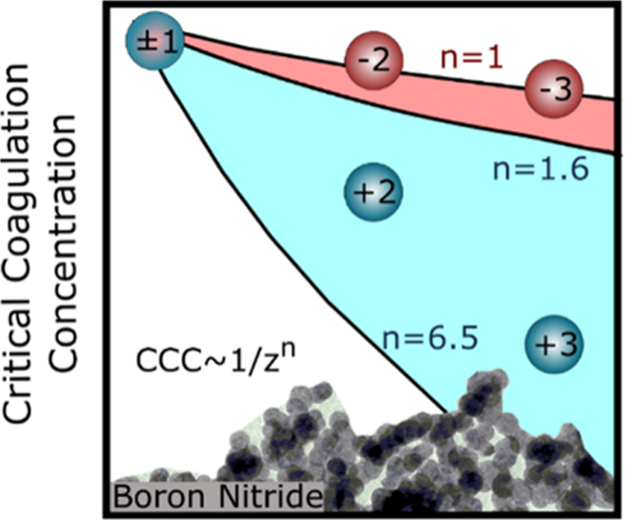

The charging and aggregation properties of boron nitride nanospheres (BNNSs) were investigated in the presence of electrolytes of different compositions and valences in aqueous suspensions. The influence of mono- and multivalent cations (counterions) and anions (coions) on the colloidal stability of the negatively charged particles was studied over a wide range of salt concentrations. For monovalent ions, similar trends were determined in the stability and charging of the particles irrespective of the salt composition, i.e., no ion-specific effects were observed. Once multivalent counterions were involved, the critical coagulation concentrations (CCCs) decreased with the valence in line with the direct Schulze–Hardy rule. The dependence indicated an intermediate charge density for BNNSs. The influence of the coions on the CCCs was weaker and the destabilization ability followed the inverse Schulze–Hardy rule. The predominant interparticle forces were identified as electrical double-layer repulsion and van der Waals attraction. These findings offer useful information to design stable BNNS dispersions in various applications, where mono- and multivalent electrolytes or their mixtures are present in the samples.

## Introduction

Boron nitride (BN) is a widely studied inorganic nanomaterial; it exists in several crystalline forms.^1^ Among them, hexagonal BN (h-BN) is of special interest since h-BN possesses a similar structure to graphene with alternating B and N atoms in a planar hexagonal lattice.^2^ The B–N covalent bond, in contrast to the C–C bonds in graphene, manifests an ionic character owing to the electronegativity of the N atoms.^3^ Two-dimensional (2D) h-BN nanomaterials have been extensively studied in the past few years.^4^ However, sheets of h-BN are able to stack into multilayers to form 3D nanostructures such as BN nanospheres (BNNSs)^5^ or wrap into BN nanotubes (BNNTs).^6^ These are promising materials; accordingly, they show distinct electronic and optical properties compared to their isoelectronic counterpart.^7^ Both BNNSs and BNNTs display outstanding features, such as high thermal stability and conductivity,^8^ chemical inertness,^9^ corrosion, and oxidation resistance.^10^

Due to the above-mentioned properties, BNNSs have been proposed in many applications, for instance, as an effective spherical catalyst support.^11−13^ It was recently shown that BNNSs possess considerable biocompatibility^6,14^ and water dispersibility^15^ compared to carbon materials; therefore, medical applications have emerged.^15,16^ For instance, targeted drug delivery has also been considered.^17,18^ The application of BN-based nanomaterials in boron neutron capture therapy (BNCT) for the treatment of cancer yielded very promising results.^19,20^ Since these materials contain a substantial number of boron atoms, BNNSs can serve as boron sources in BNCT, and the spherical shape is especially advantageous due to the large contact area with cell membrane receptors.^14^

Most of these applications rely on BN particles dispersed in a liquid medium, most frequently in an aqueous environment; therefore, the investigation of the colloidal stability of BN suspensions in different ionic media is required for their utilization in specific applications. Despite the fact that ion-specific effects have shown a significant influence on the charging and aggregation properties of different colloidal particles in aqueous samples,^21−24^ this issue has not been explored for BN dispersions so far.

In general, the classical theory developed by Derjaguin, Landau, Verwey, and Overbeek (DLVO)^25,26^ predicts the behavior of charged colloidal particles suspended in electrolyte solutions taking into account the attractive van der Waals and repulsive electric double-layer interparticle forces. Such dispersions are stable at low salt concentrations and they tend to aggregate with increasing electrolyte concentrations. The transition point between the reported slow and fast aggregation regimes is denoted the critical coagulation concentration (CCC).^27^

The DLVO theory only considers the concentration and the valence of the electrolyte constituents through the ionic strength, while the chemical nature of the ions is disregarded. Therefore, it predicts the same effect of monovalent electrolytes of different compositions on the colloidal stability. However, ions of the same valence may lead to different CCCs, as shown previously.^21−23,28,29^ The order of the CCCs in the presence of monovalent ions of different compositions can be predicted by the Hofmeister series, which classifies ions according to their hydration level and affinity to surfaces; thus, the order of CCCs differs for hydrophobic and hydrophilic particles.^29^ This series was originally developed to demonstrate the stabilization power of simple electrolytes in protein solutions;^30^ nevertheless, it was also applied for colloidal particle dispersions. However, CCCs of novel nanomaterials may differ from this sequence;^22,31^ therefore, their colloidal stability must be systematically assessed in monovalent electrolyte solutions.

The case of multivalent ions and the effect of ionic valence on particle aggregation are considered by another approach, namely, the Schulze–Hardy rule.^32^ It predicts that the CCC strongly depends on the valence of the dissolved counterions, i.e., ions of the opposite sign of charge as the particles.^33^ Moreover, it has been recently confirmed that multivalent coions, i.e., ions of the same sign as the particles, also have an impact on the CCC, although their destabilization power is weaker. This rule has been introduced in the literature as the inverse Schulze–Hardy rule.^34−36^ Both direct and inverse Schulze–Hardy rules predict a decrease of the CCC with the valence; its influence strongly depends also on the surface charge density of the particles.

Although, in some publications dealing with different biomedical applications of BNNSs, colloidal stability has been estimated through ζ-potential and particle size distribution measurements^16,18−20^ and the effect of some electrolytes on the dispersion features has been reported once for 2D h-BN,^37^ there is a lack of comprehensive studies on the charging and aggregation of colloidal BNNS particles in salt solutions of different compositions and valences. To fill this gap, in the present study, we focused on the specific ion effects on the stability of BNNS colloids. We aimed at providing an overview of the charging and aggregation features of the BNNSs synthesized in our laboratory in the presence of electrolytes, which are used in various applications.

## Experimental Methods

### Materials

Trimethyl borate (B(OMe)_3_) was purchased from ACROS Organics. NH_3_ (Linde), N_2_ (Merck), and Ar (Merck) gases were used. The salt concentration during the measurements was adjusted by analytical-grade salts such as NaCl, KCl, CsCl, MgCl_2_·6H_2_O, K_2_SO_4_, K_3_[Fe(CN)_6_] (VWR), and LaCl_3_ (Alfa Aesar), and they were used without further purification. The pH of the suspensions was adjusted by HCl (VWR) and NaOH (VWR). All measurements were carried out at 25 °C and a pH value of 7.0 ± 0.5, unless otherwise noted. High-purity water obtained from the VWR Purity TU + machine was used for all sample preparations. Water and all of the prepared salt solutions were filtered with a 0.1 μm syringe filter (Millex) prior to use to avoid dust contamination. The concentration of the BNNS particles was always kept at 5 mg/L in the light scattering measurements.

### Synthesis of BNNS

The BNNSs were synthesized by a continuously operated chemical vapor deposition (CVD) process as previously reported elsewhere,^38^ which was slightly modified to improve the material’s properties. B(OMe)_3_ was used as the B source, while NH_3_ gas served as the N source. The reaction was carried out in two stages using a quartz tube in a heating furnace, where the reactants were introduced in the gas phase. The precursor is a colorless volatile liquid of lower density than water and, thus, its vapor was transported by a carrier gas flow toward the reaction zone. In the first stage, the precursor reacted with the NH_3_ entering the furnace heated to 980 °C at a flow rate of 40.0 mL/min along with N_2_ at a flow rate of 60.0 mL/min, which served as the carrier gas, and Ar at a flow rate of 75.0 mL/min. The obtained white product from the CVD stream was collected at 5 °C. The growth mechanism and the structural transformation from an intermediate monomer to a BN layer and spherical BNNSs are predicted to happen through intermolecular eliminations and, hence, the morphology of the spherical particles depends on the elimination yield of MeO_2_ groups.^38^ The synthesis includes a second-stage annealing process of the primer product at 1100 °C, conducted in an inert atmosphere of Ar instead of using NH_3._^16^ This stage serves to improve the crystallinity of the obtained product and to reduce oxygen content.^39^ The reaction conditions needed to reach the desired morphology and size of the nanoparticles were optimized in terms of gas flow and temperature. Applying such conditions, quasi-uniform BNNSs with controlled crystallinity and good purity were successfully fabricated.

### Characterization of BNNS

**Figure 1:**
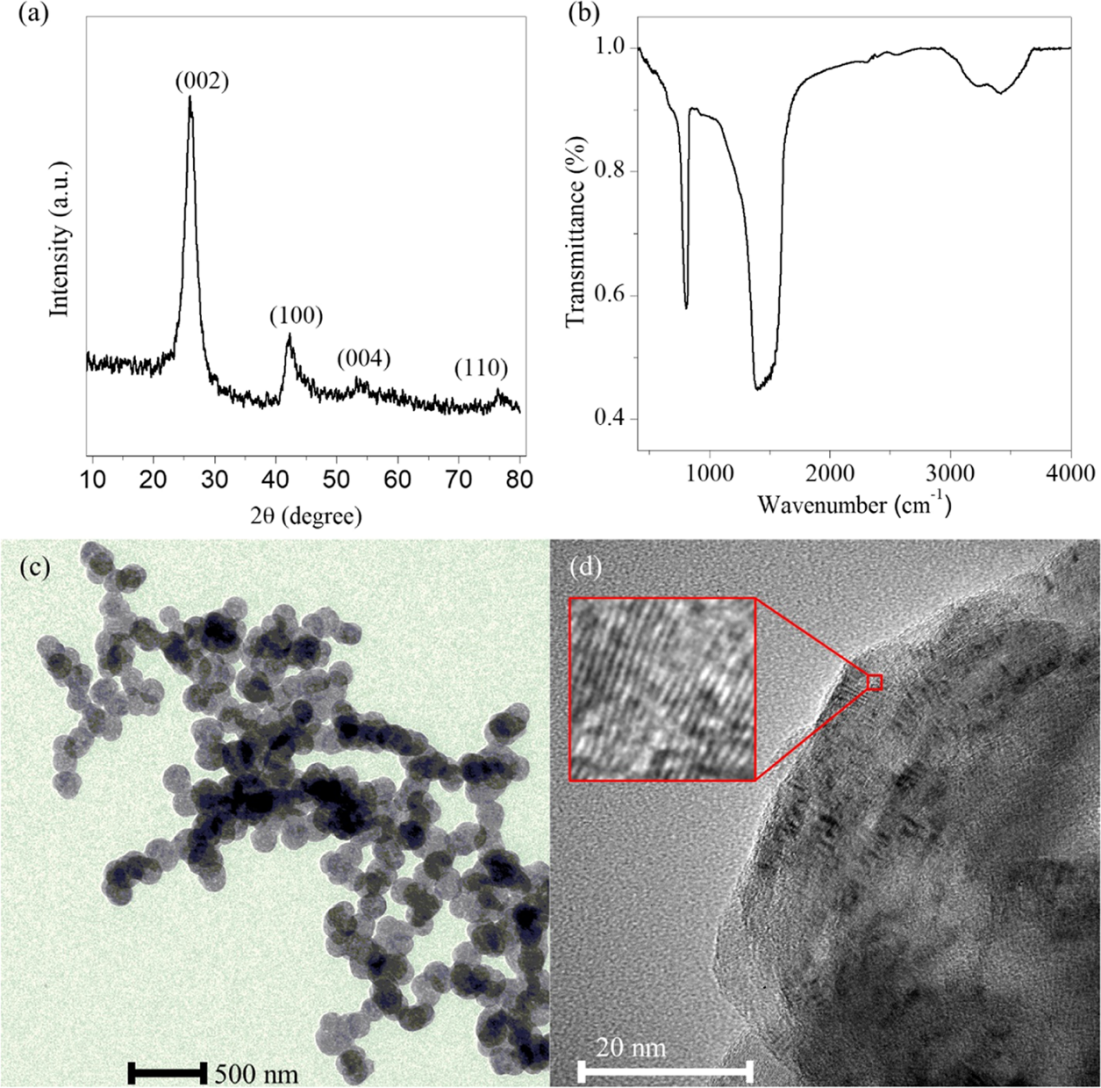
Characterization of the synthesized BNNSs. XRD diffraction pattern (a), Fourier transform infrared (FTIR) spectrum (b), TEM (c), and high-resolution TEM (HR-TEM) (d) images. In (d), the inset shows the crystal planes at 1 800 000 magnification.

Powder X-ray diffraction (XRD) measurements were conducted with a Rigaku Miniflex II desktop diffractometer using Cu Kα radiation (α = 0.15418 nm) at 40 kV accelerating voltage and 30 mA with a scan speed of 2 min^–1^. Infrared spectra were recorded by a Bruker Vertex 70 FT-IR spectrometer; the spectra were measured from 400 to 4000 cm^–1^, with a resolution of 4 cm^–1^. The morphological characteristics of the BNNSs were investigated by transmission electron microscopy (TEM) using an FEI Tecnai G^2^ 20 X Twin microscope at 200 kV accelerating voltage. The results of these measurements are presented in Figure 1.

### Electrophoresis

Electrophoretic measurements were performed with a Zetasizer Nano Instrument (Malvern), equipped with a 4 mW He–Ne laser (633 nm wavelength). The obtained electrophoretic mobility (*u*) values were converted into zeta potentials (ζ) with the Smoluchowski equation^40^
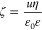
1where ε is the dielectric constant of the medium, η is the dynamic viscosity of water, and ε_0_ is the dielectric permittivity of the vacuum. The product of the inverse Debye length and the radius of the particles was 17 at 1 mM ionic strength (the lowest ionic strength used), which justifies the use of the Smoluchowski equation. For the determination of electrophoretic mobility of the particles, appropriate volumes of salt solutions and water were mixed to obtain the desired ionic strength. Then, the BNNS particles were added from the stock suspension, leading to a particle concentration of 5 mg/L and a final volume of 2 mL. The samples were allowed to rest for 2 h at room temperature before each measurement, and the equilibration time in the device was 1 min. The experiments were performed in omega-shaped plastic cuvettes (Malvern). The reported values were the average of five individual measurements, and the average error of the determined ζ-potentials is about 2–5% depending on the magnitude of the particle charge.

### Dynamic Light Scattering (DLS)

**Figure 2:**
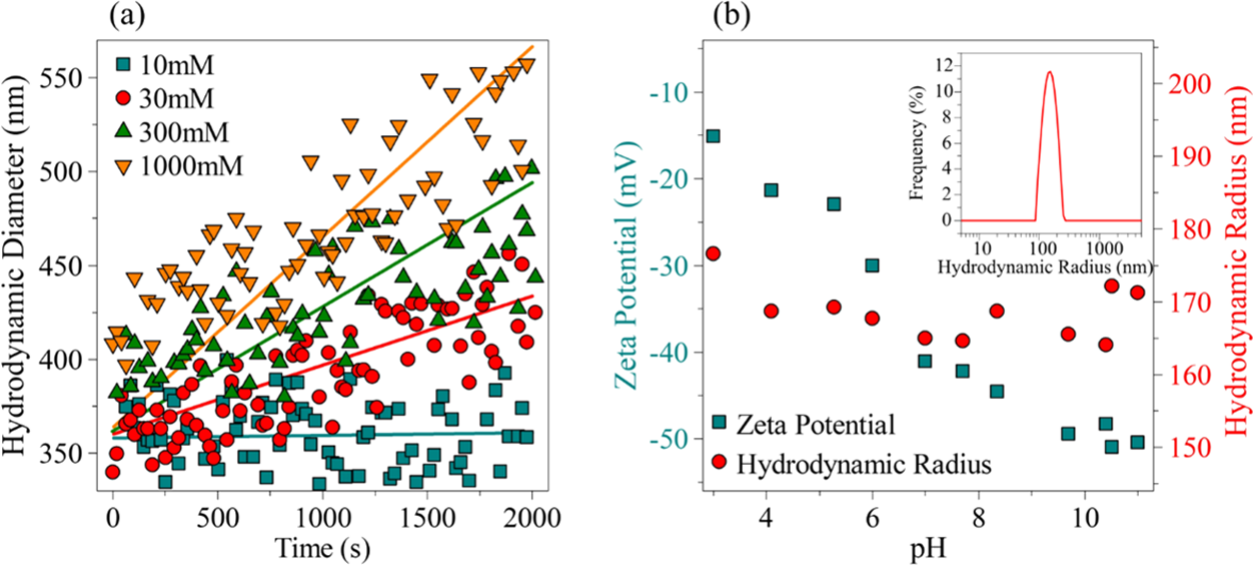
(a) Time-resolved DLS measurements at different KCl concentrations at 5 mg/L BNNS concentration. The solid lines show linear fits used to calculate the apparent aggregation rate constants, which were obtained with eq 2. (b) ζ-Potentials (squares, left axis) and hydrodynamic radii (circles, right axis) of BNNS as a function of the pH. The inset shows the intensity-weighted size distribution of BNNS at pH 7. The measurements were performed at 1 mM ionic strength, and the standard deviations of the hydrodynamic radius and ζ-potential data were 5 nm and 3 mV, respectively.

Time-resolved dynamic light scattering (DLS) measurements were performed to assess the colloidal stability of the samples by observing the early stages of aggregation.^41,42^ The experiments were carried out using a Zetasizer Nano Instrument (Malvern) in backscattering mode at 173° scattering angle. The apparent hydrodynamic radius (*R*_h_) was calculated by the Stokes–Einstein equation from the translational diffusion coefficient, which was extracted from the correlation function by the second-order cumulant fit. The correlation function was recorded for 20 s, and the time evolution of the hydrodynamic radius was followed over 100 consecutive measurements. Typical results of these measurements, where the variation of the hydrodynamic diameter was observed with time at different KCl concentrations, are shown in Figure 2a. The initial rate of increase in size was used to calculate the apparent aggregation rate coefficients (*k*_app_) as^43^
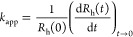
2where *R*_h_(0) is the hydrodynamic radius of the primary particles measured in stable suspension and *t* is the time of the experiment. Note that the initial hydrodynamic radius was higher than the geometrical radius due to the presence of some aggregates in the dispersions and also that the increase was studied in the time span in which the radius changed linearly with time. The slope depended on the salt concentration; at higher ones, the slope is larger, indicating that the dispersions are destabilized, and thus rapid aggregation occurs. For each measurement, 2 mL dispersions were prepared similar to that described above for electrophoresis, with the exception that the DLS measurements were commenced by adding the desired volume of particle stock dispersions to the solutions containing all other components. Furthermore, the samples were equilibrated for 60 s in the instrument prior to the measurements. This protocol led to a mean error of 5%.

## Results and Discussion

First, the synthesized BNNSs were characterized by different techniques to verify the successful formation of the product and to define its morphological characteristics. Then, charging and aggregation properties of the nanospheres were investigated in the presence of different electrolytes by electrophoresis and DLS. Note that the experimental conditions (e.g., pH and salt and BNNS concentrations) in a given suspension were the same in both types of measurements. The results were interpreted within the framework of the DLVO theory, the Hofmeister series, and the Schulze–Hardy rule.

### Characterization of the BNNSs

The XRD diffraction pattern presented in Figure 1a revealed well-separated broad peaks at 2θ 25.92 and 42.34°, corresponding to the (002) and (100) crystal planes, respectively. All of the peaks can be attributed to the hexagonal BN, in agreement with that previously reported for h-BN.^38^ The characteristic peaks corresponding to the (004) crystal plane^44^ can also be seen at 53.37° and the (110) plane^45^ at 76.29°. No other obvious diffraction peaks can be observed, which indicates the high purity of the prepared material. To further verify the successful synthesis, FTIR spectroscopy was employed. The spectrum (Figure 1b) contained the typical absorption bands at 806 and 1401 cm^–1^ representing the out-of-plane B–N–B bending and in-plane B–N stretching vibration modes, respectively.^38^ Besides, absorption bands can be observed at 3209 and 3415 cm^–1^, which can be assigned to the asymmetric stretching of N–H groups and O–H stretching of hydroxyl groups.^16^ The synthesis of the particles was confirmed also with TEM images by exploring the morphology and the dimensions of the nanoparticles. The images show clearly visible spherical particles with an average diameter of 110 nm and quasi-uniform size distribution (Figure 1c). On the HR-TEM image (Figure 1d), the crystal planes were well observed after magnification, and these results further indicate that the obtained BNNSs possess good crystallinity. The determined lattice plane spacing from the TEM image was 3.476 Å, whereas 3.429 Å was calculated for the (002) crystal plane using Bragg’s law.

The pH-dependent charge and size variations were investigated in the pH range of 3–11 by electrophoresis and DLS, respectively (Figure 2b). The BNNSs exhibit a negative charge, owing to the abundance of the deprotonated hydroxyl groups under these conditions.^46^ The ζ-potential values indicate that the particles have a relatively high magnitude of surface charge over a wide range of pH. With increasing pH, the particles acquire a greater negative charge. Further experiments were conducted at pH 7. Under this condition, the particles possess a considerable negative charge (−41 mV ζ-potential), predicting high colloidal stability. In Figure 2b, the measured hydrodynamic radii at different pH values are also presented. However, a clear tendency could not be observed, as the particles maintained their size while varying the pH value of the dispersions. At pH 7, the hydrodynamic radius was obtained as 165 ± 5 nm, and the polydispersity index was 0.27 ± 0.02. The intensity-weighted distribution of the hydrodynamic radius is shown in the inset of Figure 2b. Note that the hydrodynamic (DLS) and geometrical (TEM) sizes are considerably different. This is due to the presence of some aggregates in the dispersions, which contribute to the scattered intensity to a large extent, giving rise to higher hydrodynamic radii.

### Colloidal Stability in the Presence of Monovalent Salts

Aggregation rates and ζ-potentials of BNNS particles were measured in different ionic environments. The colloidal stability of the suspensions was assessed in time-resolved DLS measurements. The rate of aggregation in the samples was expressed in terms of the stability ratio (*W*) as^43,47^
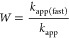
3where the fast subscript indicates the fast aggregation of the particles, where the aggregation is diffusion controlled. The *k*_app(fast)_ value was determined separately in all of the investigated systems in the fast aggregation regime and used to calculate the stability ratios. Considering eq 3, it can be easily realized that higher stability ratios are measured for slow particle aggregation, while the values close to unity indicate rapidly aggregating particles.

**Figure 3:**
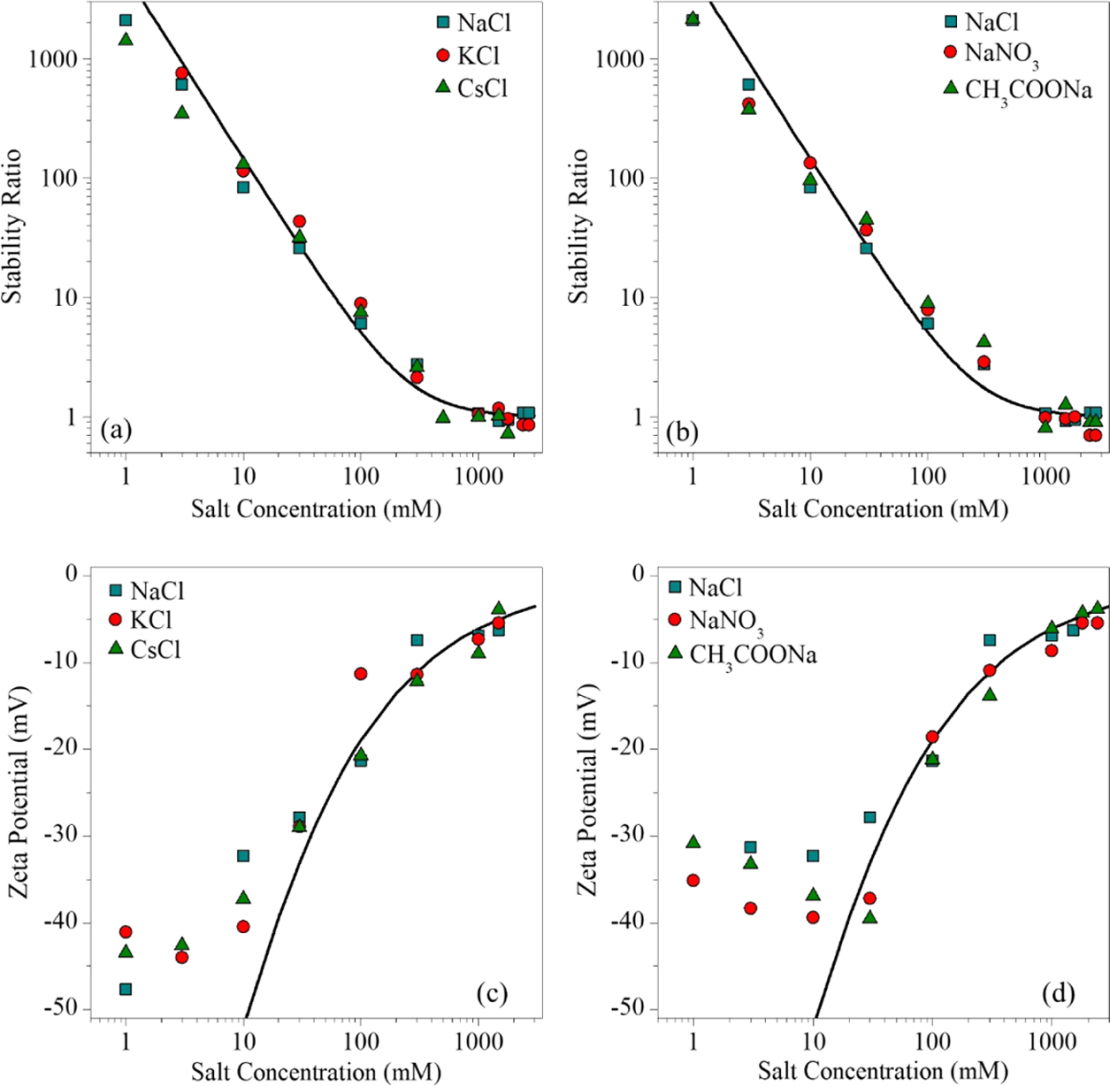
Stability ratios of BNNS particles in the presence of monovalent counterions (a) and coions (b) and the corresponding ζ-potentials (c) and (d), respectively. The solid lines in (a) and (b) were calculated using eq 4, whereas eq 6 was used in (c) and (d). The uncertainty of the stability ratio data is about 5%.

First, we investigated the effect of monovalent counterions (Na^+^, K^+^, Cs^+^) and coions (Cl^–^, NO_3_^–^, CH_3_COO^–^) of the negatively charged BNNSs. The determined stability ratios at different electrolyte concentrations are presented in Figure 3a,b. The general trend of the stability ratios was the same in both cases. Accordingly, slow aggregation can be observed at low salt concentrations, as in this region, the values of the stability ratios are high. With increasing salt concentration, the stability ratio decreases until it reaches unity and remains at this value thereafter. This behavior is in line with the DLVO theory.^26,48^ Accordingly, at low ionic strength, the overlap of the electrical double layers surrounding the particles gives rise to repulsive forces; therefore, the dispersions are stabilized. However, on increasing the salt concentration, the electrostatic repulsion weakens upon salt screening and, thus, rapid aggregation of the particles occurs due to predominating attractive van der Waals forces. The transition between these two regimes is the so-called critical coagulation concentration (CCC), and the aggregation tendencies in the different systems can be well quantified by this parameter, which was determined from the stability curves as follows^47^
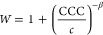
4where *c* is the molar concentration of the salts and β can be obtained from the slope of the stability ratios in the slow aggregation regime of the stability ratio versus salt concentration plot as
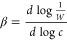
5

In the presence of monovalent salt constituents, the measured stability curves were identical within the experimental error and, thus, the onset of the rapid particle aggregation was located at the same CCC for all systems, namely, around 0.25 M, irrespective of the type of monovalent salts.

The charging features of the BNNS particles were also investigated in the presence of monovalent counterions (Figure 3c) and coions (Figure 3d) by measuring the ζ-potentials under the same experimental conditions as those for the time-resolved DLS measurements. In general, the shape of the plots was identical in all cases; the ζ-potential values became less negative as the concentration of the salts increased due to double-layer compression resulting from charge screening. Note that the values remained negative in the whole range investigated. The ζ-potential is positioned at the slip plane, determining the distance from the surface, where a hydrodynamically stagnant layer is present and moves together with the particle upon application of an external electric field.^40^ Therefore, one of the most important parameters, which can be estimated from the ζ-potentials, is the charge density at the slip plane (σ) of the particles, and it was calculated by the Debye–Hückel model as^49^
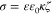
6where κ is the inverse Debye length, which includes the contribution of the ionic species to the electrical double layer.^40^ The obtained charge density values were the same within the measurement error (around −14 mC/m^2^), regardless of the type of the ions, indicating the absence of ion-specific adsorption. Considering the strong dependence of the CCC and charge density of inorganic nanoparticles on the composition of the salts,^22,23,31^ the above aggregation and charging results are rather surprising.

### General Trends in the Presence of Multivalent Salts

**Figure 4:**
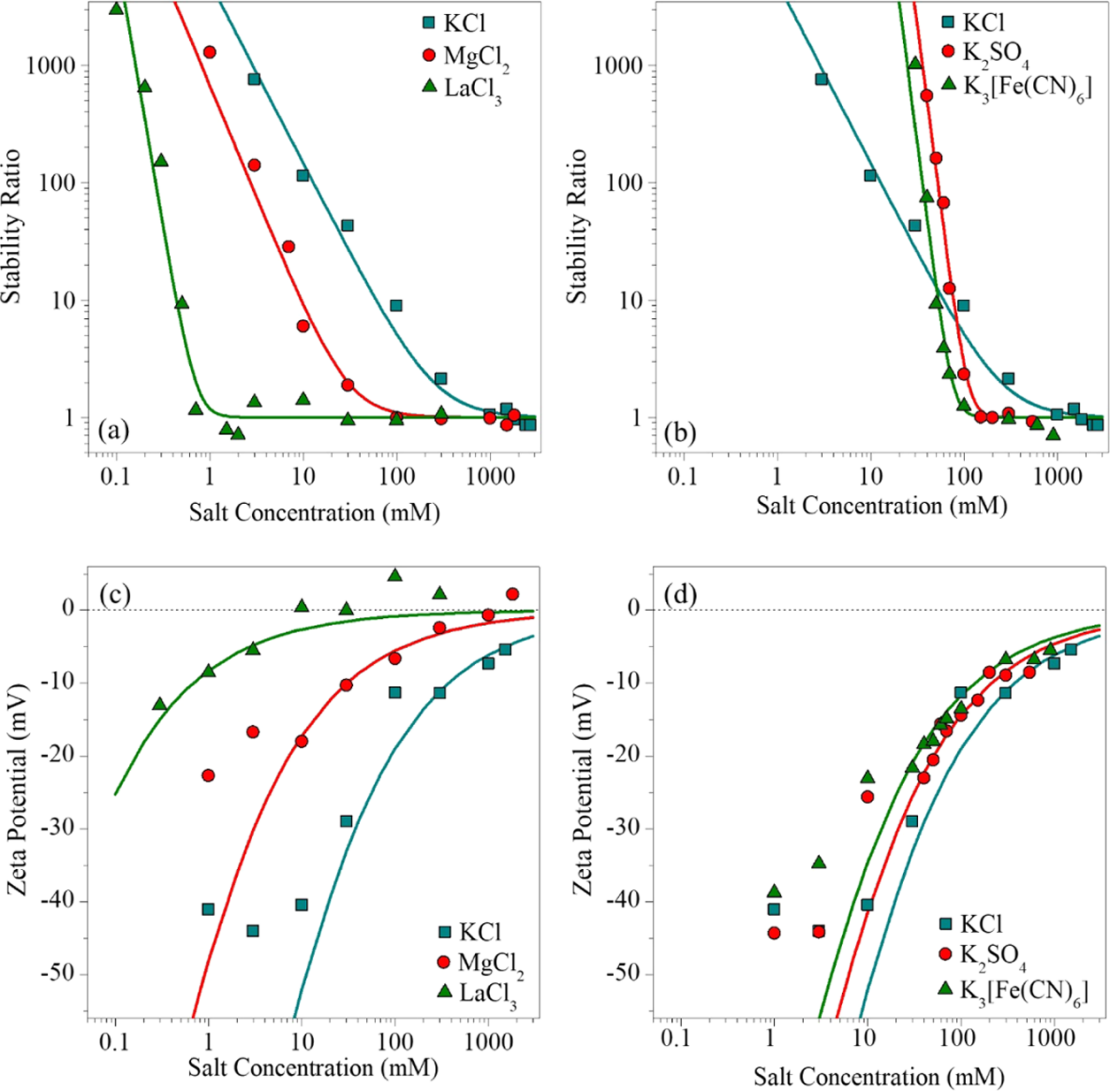
Stability ratios of BNNS particles in the presence of multivalent counterions (a) and coions (b) and the corresponding ζ-potentials (c) and (d), respectively. The solid lines in (a) and (b) were calculated using eq 4, whereas eq 6 was used in the case of (c) and (d).

Charging and aggregation properties of the BNNS particles were studied in solutions of multivalent counterions (Mg^2+^ and La^3+^) and coions (SO_4_^2–^ and [Fe(CN)_6_]^3–^) as well. The stability curves shown in Figure 4a for the multivalent counterions illustrate the characteristic slow and fast aggregation regimes, similar to the monovalent systems discussed earlier. However, for di- and trivalent ions, the CCCs are substantially lower compared to the ones determined in the presence of monovalent counterions. The CCC values decrease with increasing valence, in good qualitative agreement with the Schulze–Hardy rule.^32,50^ The quantitative interpretation of the CCCs in terms of this theory will be discussed later. Then, we investigated the effects of multivalent anions on the colloidal stability of negatively charged BNNS particles (Figure 4b). One can observe that the CCC shifts toward lower salt concentrations with increasing valence of the coion, but the shift is much smaller than that in the case of multivalent counterions. The CCC values for multivalent ions are presented in Table 1.

**Table 1:** Characteristic Aggregation and Charging Data of the BNNS Particles Obtained in the Presence of Mono- and Multivalent Salt Constituents

salt	KCl	MgCl_2_	LaCl_3_	K_2_SO_4_	K_3_[Fe(CN)_6_]
CCC (mM)^a^	250	30	0.7	110	75
σ (mC/m^2^)^b^	–14	–4	–0.6	–11	–9
ζ (mV)^c^	–8.6	–10.0	–9.9	–13.7	–13.4

a Critical coagulation concentration was calculated by eq 4. The error of CCC determination is about 10%.

b Charge density was calculated using eq 6.

c ζ-Potentials at the CCC.

In Figure 4c,d, the charging features of the particles are represented, while varying the counterion and coion valences, respectively. The magnitudes of the ζ-potentials at a given salt concentration followed the monovalent > divalent > trivalent order in the case of counterions and coions as well. The surface charge densities were extracted from the salt concentration-dependent potential data by eq 6. The values (Table 1) decreased in the order of K^+^ > Mg^2+^ > La^3+^ for the cations and Cl^–^ > SO_4_^2–^ > [Fe(CN)_6_]^3–^ in the case of anions. Note that the extent of this decrease is significantly smaller for the coions. Comparing the charge density values for monovalent and multivalent counterions, the multivalent ions were much more effective in reducing the ζ-potential of the particles due to their ability to adsorb more effectively to the particle surface, which was also reported for other particles dispersed in solutions of multivalent ions.^22,33,34,41^

Let us now interpret the above detailed experimental results concerning the influence of salt composition and valence of the ions on the colloidal stability of BNNS particles in terms of the Hofmeister series and the Schulze–Hardy rule. The CCC is the most appropriate parameter to quantify the deviation of the aggregation features for different systems; therefore, its tendencies will be discussed by varying the type of electrolytes.

### Dependence of the CCC on the Composition of Monovalent Electrolytes

As mentioned above, the CCC values were the same (0.25 M) within the experimental error for all monovalent salts, irrespective of the composition, i.e., for both counterions and coions. This behavior is atypical for colloidal particles; however, some exceptions have already been reported. Accordingly, aggregation of highly charged latexes was not sensitive to the type of ions.^33^ Furthermore, polymer-functionalized colloid particles were also insensitive to the chemical composition of surrounding monovalent electrolytes,^51^ in agreement with the DLVO theory, which considers only the ionic strength, i.e., the valence and concentration of the ions present in the system. The BNNSs showed the same behavior, and this feature may be explained as follows. First, the particles are of high negative charge under the conditions applied and, hence, the small extent of ion adsorption did not give rise to a significant change in the charge densities and in the strength of the double-layer forces. Second, the lamellar structure of BNNSs may allow diffusion of ions into the interlamellar space and, thus, the interfacial counterion concentration can be lower than expected. Due to the lower concentration, the electrical double layers remain strong independent of the type of ions, leading to stable dispersions up to elevated ionic strength. However, we do not have direct experimental evidence for the ion diffusion into the interlamellar space. Again, such an absence of the ion-specific effect on the colloidal stability of the BNNSs is rather unusual. However, it can be beneficial in applications in which the particles are dispersed in monovalent electrolyte mixtures and the goal is to obtain stable colloids.

### Dependence of the CCC on the Valence

As discussed before, multivalent ions are more powerful in destabilization of BNNS suspensions. It was shown earlier that the trend in CCC depends on the sign and valence of the ions present; therefore, the influence of counterions and coions should be treated differently.^35^

In the case of counterions, the CCC decreases in the order K^+^ > Mg^2+^ > La^3+^ (Table 1). The types of salts are not directly considered in the DLVO model; however, this theory takes into account the valence of ions. The effect of the ionic valence is described by the Schulze–Hardy rule,^50,52^ which states that the CCC of colloidal particles strongly depends on the ionic valence (*z*) of the dissolved ions, and the dependence can be quantified as
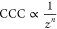
7where *n* can vary between 1.6 and 6.5 depending on the surface charge density of the particles. For asymmetric electrolytes containing multivalent counterions, the dependence can be described for particles with a low surface charge by *n* = 1.6 in eq 7,^34^ while for highly charged particles, a stronger dependence (*n* = 6.5) is predicted.^35^ However, this strong decrease of the CCC with the valence can be derived from the DLVO theory only for surfaces of unrealistically high magnitude of charge.

**Figure 5:**
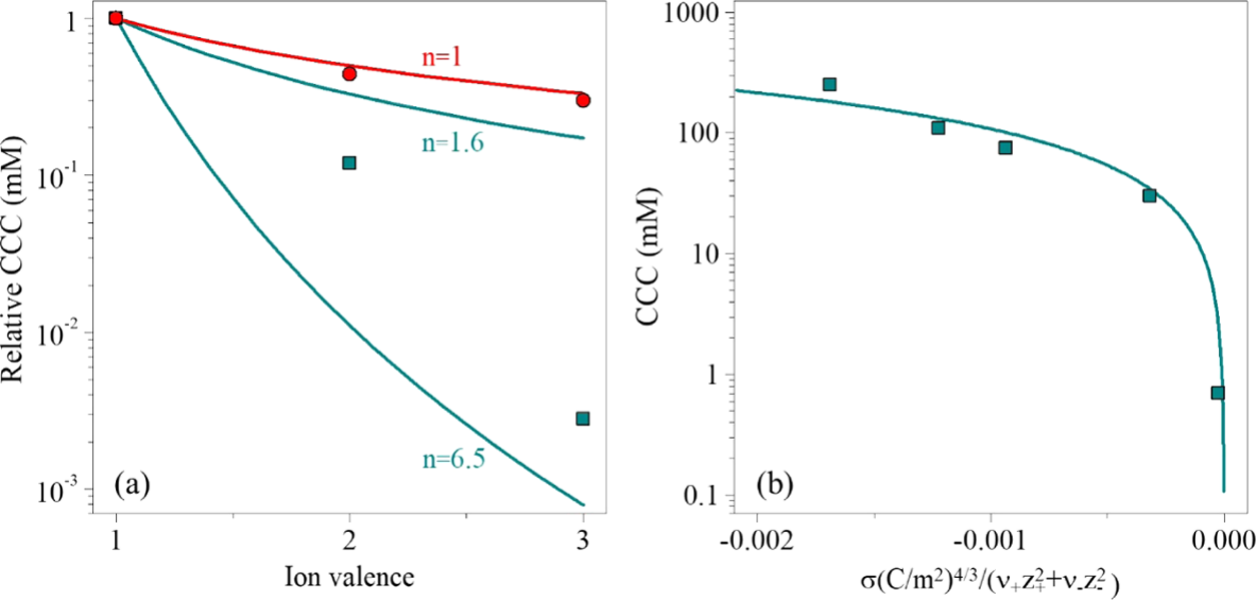
(a) Relative CCC values (normalized to the CCC obtained in the presence of KCl) as a function of different counterion (squares) and coion (circles) valences. The solid lines indicate the direct (for *n* = 1.6 and 6.5 in eq 7) and the inverse (*n* = 1 in eq 7) Schulze–Hardy rules. (b) Dependence of the CCC on the charge density at the slip plane, which was normalized with the stoichiometry and the valence of the electrolytes. The solid line was calculated with eq 8.

Figure 5a shows the relative CCCs normalized to the value in the presence of KCl salt for all salts investigated in the present study together with the expected CCCs from the Schulze–Hardy rule (eq 7), with both *n* values indicated as the upper and lower limits of CCCs. All our obtained data appear below the upper limit of the *z*^–1.6^ dependence, while the lower limit (*z*^–6.5^) overestimates the effect for multivalent ions, indicating that BNNS particles possess intermediate surface charge density.

The obtained charge density values (Table 1) demonstrate that the magnitude of this parameter strongly decreases with increasing valence, since the multivalent counterions adsorb stronger than monovalent ones to oppositely charged substrates and, thus, they progressively reduce the charge density. This reduction leads to weaker double-layer forces and becomes more pronounced with increasing valence, and thereby leads to a lower CCC.

Nevertheless, the ions of the same sign of charge as the particles show a trend in the CCC values as Cl^–^ > SO_4_^2–^ > [Fe(CN)_6_]^3–^ (Table 1). Multivalent coions hardly adsorb on like-charged particles, and hence the particle is expected to remain highly charged.^53^ When multivalent ions are the coions, the approach of the inverse Schulze–Hardy rule was proposed, which states that the dependence of the CCC on coion valence is not as significant (*n* = 1 in eq 7)^34,50^ as in the case of oppositely charged multivalent ions. This dependence is also presented in Figure 5a and it is in good quantitative agreement with the CCC values obtained from the aggregation experiments with different coions.

### Predominating Interparticle Forces

Based on the data presented in Table 1, one may notice that the values of charge density and the ζ-potential (at the CCC) follow the same order in the case of multivalent ions as the CCCs. In the case of monovalent ions, the same charge was determined for all systems within the experimental error, since the measured ζ-potential values were about the same. This confirms that the type of monovalent electrolytes does not influence the charge of BNNSs and, thus, ion-specific effects are absent. For multivalent ions, the obtained surface charge is the lowest for the La^3+^ ion, and hence, the repulsive electrical double-layer forces are the weakest in this case. Therefore, destabilization occurs at the lowest CCC. A similar effect could be observed for coions as well, where the [Fe(CN)_6_]^3–^ causes the lowest CCC; however, the destabilizing power here originates from the contribution to the ionic strength, i.e., trivalent ions increase the ionic strength more significantly than mono- and divalent ones.

To further explore the origin of the interparticle forces responsible for the stability of the BNNS suspensions, a relation derived from the DLVO theory was used to calculate the CCC values from the obtained surface charge densities as^24,54^
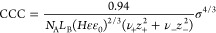
8where *N*_A_ is Avogadro’s number, *L*_B_ is the Bjerrum length, which is 0.72 nm at room temperature in water,^21^ ν_+_ and ν_–_ are the stoichiometric coefficients of the cation and anion, respectively, *z*_+_ and *z*_-_ represent the cation and anion valences, respectively, and *H* is the Hamaker constant. To achieve the best agreement between the calculated and measured values, a Hamaker constant of 4.0 × 10^–21^ J was applied in eq 8.

The CCCs at different charge densities together with the fit obtained from eq 8 are presented in Figure 5b. The relatively good agreement between the experimental and the calculated data confirms that the predominating interparticle forces are of DLVO origin since only DLVO-type forces were included in the calculations to derive eq 8. However, the ion-specific interactions also play an important role through the extent of the ion adsorption for the multivalent counterions, which leads to different surface charge densities, and thus significant deviation in the strength of the repulsive double-layer forces. Accordingly, multivalent counterions destabilize particle suspensions more effectively than the monovalent ones due to their higher affinity to oppositely charged surfaces. Therefore, the adsorption processes and related charging properties depend strongly on the valence of counterions and coions as well; nevertheless, the predominating interparticle forces are of DLVO origin. This fact is further confirmed by the agreement between the CCC tendencies with the direct and inverse Schulze–Hardy rules.

## Conclusions

The effect of salts composed of monovalent counterions (Na^+^, K^+^, Cs^+^) or coions (Cl^–^, NO^3–^, CH_3_COO^–^) and multivalent counterions (Mg^2+^, La^3+^) or coions (SO_4_^2–^, [Fe(CN)_6_]^3–^) on the aggregation and charging features of BNNS particles was investigated for the first time by electrophoresis and dynamic light scattering techniques. Similar trends and CCC values were determined for monovalent ions of both charges; therefore, no ion-specific effect was observed. The ζ-potentials also showed the same tendencies with increasing salt concentration and, thus, the same surface charge density was determined in the presence of monovalent salt constituents irrespective of the electrolyte composition. This behavior is in striking contrast to most of the results reported earlier for nanoparticles or colloidal particles of various compositions.

Concerning the effect of the ionic valence, significant differences in the obtained CCC values were observed. This phenomenon was explained by the Schulze–Hardy rule for counterions since the order of CCCs decreased as
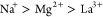
The coions did not show as significant an effect on the CCC by varying their valence as shown by the counterions; however, a weak dependence is still present in line with the inverse Schulze–Hardy rule. The order of ions with decreasing CCC values was determined as follows
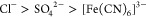
Comparison of the CCCs obtained from the experiments to the ones calculated by the DLVO theory revealed that the interparticle forces originate from the interplay between the repulsive electrical double-layer and attractive van der Waals forces, as predicted by the DLVO theory. The variation of the CCC in the systems is the result of the different absorption affinity and the different contribution to the ionic strength when mono-, di-, and trivalent ions are present. No similar identification of interparticle forces has been reported for BNNSs so far.

Through the comprehensive set of CCC values, these results provide new knowledge that will be useful in further investigations of BNNS dispersions containing electrolytes or their mixtures. Finally, the surprising differences in the effect of mono- and multivalent ions on the dispersion characteristics of BNNSs shed light on the need for detailed colloidal stability studies once the nanoparticles are applied, for instance, in biomedical treatments, in which different electrolyte components of the biofluids may play an important role in the destabilization of BNNS.
